# Quantitative Proteomic Analysis of Venoms from Russian Vipers of *Pelias* Group: Phospholipases A_2_ are the Main Venom Components

**DOI:** 10.3390/toxins8040105

**Published:** 2016-04-12

**Authors:** Sergey I. Kovalchuk, Rustam H. Ziganshin, Vladislav G. Starkov, Victor I. Tsetlin, Yuri N. Utkin

**Affiliations:** Shemyakin-Ovchinnikov Institute of Bioorganic Chemistry, Russian Academy of Sciences, Moscow 117997, Russia; xerx222@gmail.com (S.I.K.); rustam.ziganshin@gmail.com (R.H.Z.); vladislavstarkov@mail.ru (V.G.S.); vits@mx.ibch.ru (V.I.T.)

**Keywords:** snake venom, viper, *Vipera kaznakovi*, *Vipera nikolskii*, *Vipera orlovi*, *Vipera renardi*, proteome, mass-spectrometry

## Abstract

Venoms of most Russian viper species are poorly characterized. Here, by quantitative chromato-mass-spectrometry, we analyzed protein and peptide compositions of venoms from four *Vipera* species (*V. kaznakovi*, *V. renardi*, *V. orlovi* and *V. nikolskii*) inhabiting different regions of Russia. In all these species, the main components were phospholipases A_2_, their content ranging from 24% in *V. orlovi* to 65% in *V. nikolskii*. Altogether, enzyme content in venom of *V. nikolskii* reached ~85%. Among the non-enzymatic proteins, the most abundant were disintegrins (14%) in the *V. renardi* venom, C-type lectin like (12.5%) in *V. kaznakovi*, cysteine-rich venom proteins (12%) in *V. orlovi* and venom endothelial growth factors (8%) in *V. nikolskii*. In total, 210 proteins and 512 endogenous peptides were identified in the four viper venoms. They represented 14 snake venom protein families, most of which were found in the venoms of *Vipera* snakes previously. However, phospholipase B and nucleotide degrading enzymes were reported here for the first time. Compositions of *V. kaznakovi* and *V. orlovi* venoms were described for the first time and showed the greatest similarity among the four venoms studied, which probably reflected close relationship between these species within the “kaznakovi” complex.

## 1. Introduction

Venomous snakes inhabit all continents of the globe except Antarctica. They are particularly abundant in tropical areas of Asia, Africa, South America and Australia. Russia, despite its large territory, is inhabited by only a small number of poisonous snake species, which belong to three genera: *Gloydius*, *Macrovipera* and *Vipera*. The *Vipera* genus is the most speciose in Russia and includes more than ten species, the systematics within this genus being constantly updated [1,2]. The most abundant species is common (or European) adder *Vipera berus*, which has a very large habitat in Russia, ranging from its western borders to Sakhalin and the Ussuri region. *V. berus* is also spread throughout Europe—between 68 and 45 degrees north latitude. The venom of this species is fairly well studied. Biological activities of this venom were characterized and proteolytic, fibrinolytic, anticoagulant, and phospholipolytic ones were demonstrated by *in vitro* experiments [3]. Several toxic proteins were isolated from *V. berus* venom, including phospholipase A_2_ (PLA2) [4], metalloproteinase (SVMP) [5], l-amino acid oxidase (LAAO) [6] and several others. Recently, we have partially characterized the steppe viper *V. renardi* venom, the PLA2s and Kunitz type protease inhibitors were isolated from this venom and sequenced [7]. The isolated PLA2s were studied in more details and found to exert their action both on lipid membranes [8] and on nicotinic acetylcholine receptor [9]. The venom of Nikolsky’s viper was also partially characterized and several proteins including heterodimeric neurotoxic PLA2s were identified [10,11]. The venoms of other Russian viper species are characterized very poorly. Thus, for Caucasian viper *V. kaznakovi* and Orlov’s viper *V. orlovi*, only the toxicity of venoms to insects was determined [12]. Here, we used proteomic chromato-mass-spectrometry analysis to obtain more detailed information on the composition of Russian viper venoms.

Modern proteomic analysis allows both qualitative and quantitative characterization of the venom proteins, leading to suggestions about venom biological effects. So far, among *Vipera* genus, the venoms of only three species, *i.e.*, *V. ammodytes*, *V. anatolica* and *V. raddei*, were thus studied [13,14,15]. Semi-quantitative venom analysis of *V. anatolica* showed that the most abundant toxin family was SVMPs (41.5%), followed by two cysteine-rich secretory protein (CRISP) isoforms (15.9%); other proteins represented less than 10% per family [13]. SVMPs (31.6%) were also the most abundant in *V. raddei* venom, followed by PLA2s (23.8%), and, again, the contents of other toxin families did not exceed 10% each [14]. There is no quantitative analysis of the *V. ammodytes* venom, however monomeric and heterodimeric Group II PLA2s; serine proteinases (SVSPs); Group I, II, and III SVMPs; l-amino acid oxidases (LAAOs); CRISPs; disintegrins (Dis); and growth factors were found [15]. On the whole, the above data indicate that the composition of different viper venoms might be different. It should also be noted that *V. raddei* in some publications is classified as *Montivipera raddei* and attributed to *Montivipera* genus [16], thus some differences might be attributed to the discrepancy in classification. Using quantitative proteomic, we have studied the venoms from four *Vipera* species (*V. kaznakovi*, *V. nikolskii*, *V. orlovi* and *V. renardi*) that inhabit different regions of Russia. In contrast to the venoms of earlier studied *Vipera* species where the SVMP were found to be predominant [13,14,15], we have observed that the main components of the venoms studied are PLA2s, the content of which ranged between 24 and 65%.

## 2. Results

### 2.1. Venom Proteins Identification

In this work, venom proteomes and peptidomes for four species of *Vipera* snakes were analyzed. Venom proteomes were analyzed by LC-MS/MS after in-solution trypsin proteolysis. In total, for the four *Vipera* species venoms, the search against the Serpentes database resulted in the identification of 210 proteins (Table 1 and Table 2, and Tables S1 and S2): 116 proteins were identified in *V. kaznakovi*, 124 in *V. renardi*, 135 in *V. orlovi* and 111 in *V. nikolskii* venoms. Most proteins could be matched to previously reported snake toxins. To minimize individual variations, venoms from several individual animals were pooled for analysis [12].

The proteins were categorized into 14 known venom protein families (Table 2). The most numerous classes were PLA2, SVMP, C-type lectin like (CTL) and serine protease (SP). Eleven families were represented in all viper venoms, while disintegrins (Dis) were absent in *V. nikolskii*. There were no Kunitz type proteinase inhibitors in *V. kaznakovi*, no hyaluronidase (Hya) in *V. renardi* and no bradykinin potentiating and C-type natriuretic peptides (B-NAP) in *V. renardi* and *V. nikolskii*. Besides, in the *V. nikolskii* venom, a single low abundance protein was identified belonging to Cysteine Proteases (CP), which are not common for snake venoms. Along with venom proteins, several Blood Proteins (BP) (up to 0.15% of the total protein abundance) and proteins with unclear family annotation (Other Proteins (OP)) (up to ~2% of the total protein content) were also found.

**Figure 1:**
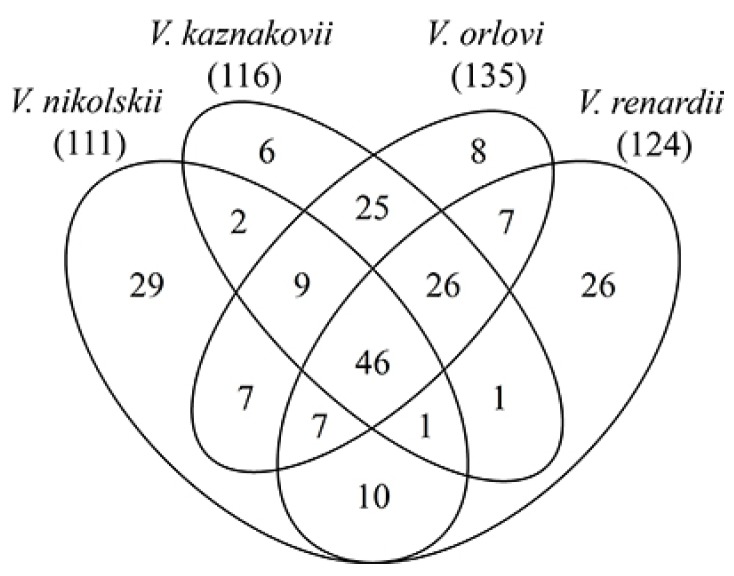
The number of common proteins in four *Vipera* species studied. The number in bracket under each species name indicates the total number of proteins identified in this species venom.

While the most numerous venom protein families were fairly similar in all snakes studied, individual protein composition was quite different (Figure 1). From 210 proteins only 46 were common for all four species and each species featured unique proteins: six in *V. kaznakovi*, 26 in *V. renardi*, eight in *V. orlovi* and 29 in *V. nikolskii*. These differences did not correlate with the total number of individual proteins identified in each venom.

### 2.2. Composition of Russian Viper Venoms

**Figure 2:**
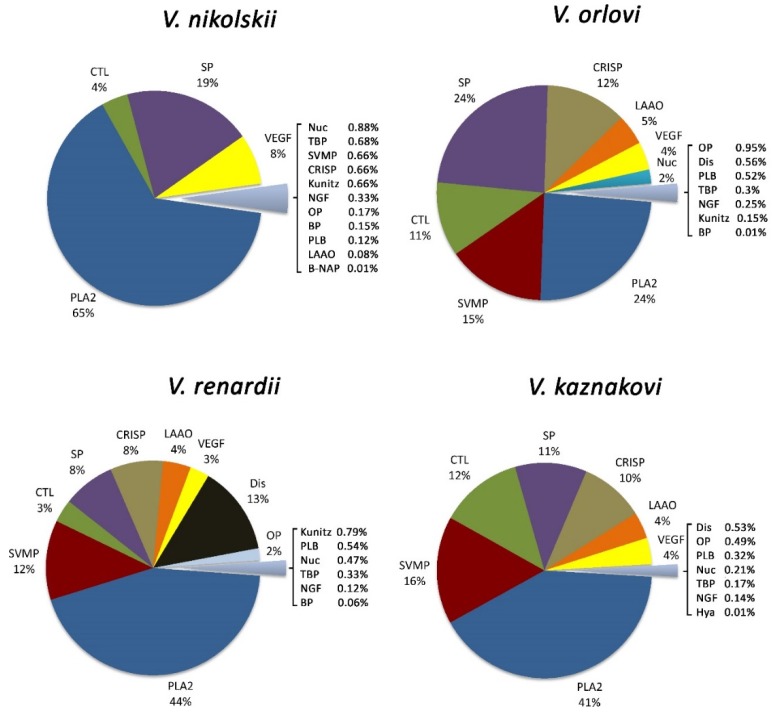
Relative abundance of venom proteins that were identified by LC MS/MS in Russian viper venoms. B-NAP: Bradykinin potentiating and C-type natriuretic peptides; BP: Blood protein; CRISP: Cysteine-rich secretory protein; CTL: C-type lectin like; Dis: Disintegrin; Hya: Hyaluronidase; Kunitz: Kunitz type proteinase inhibitor; LAAO: l-amino acid oxidase; NGF: Nerve growth factor; Nuc: Nucleic acid degrading enzymes; OP: Other protein; PLA2: Phospholipase A_2_; PLB: Phospholipase B; SP: Serine proteinase; SVMP: Metalloproteinase; TBP: Toxin biosynthesis proteins (including aminopeptidases); VEGF: Vascular endothelial growth factor.

As a result of venom protein quantification, it was found that the main venom components were PLA2s; their content ranged from about 24% in *V. orlovi* venom to more than 60% in *V. nikolskii* (Table 2, Figure 2). The overwhelming majority of PLA2s belonged to D49 subgroup of group IIA as it might be expected for the snakes from Viperidae family. The venom of *V. nikolskii* contained PLA2s only from this group. One PLA2 of S49 subgroup was highly represented in *V. renardi*. One PLA2 of group IA was observed in small amounts in three venoms and a low quantity of group IIE PLA2 was detected in *V. renardi* venom.

Altogether, the enzyme content in venom of *V. nikolskii* reached about 85%, however this venom was characterized by a very low content of SVMPs (less than 1%) and LAAO (less than 0.1%). PLA2s accounted for more than 40% in *V. kaznakovi* and *V. renardi* venoms. While the content of SVMPs was less than 1% in the *V. nikolskii* venom, they comprised 12%–16% in *V. kaznakovi*, *V. orlovi* and *V. renardi.* The highest content of SPs was in *V. orlovi* venom (24%) and the lowest in *V. renardi* (8%). LAAO was at the level of 4%–5% in all the analyzed venoms with the exception of *V. nikolskii*. Nucleic acid degrading enzymes (Nuc) represented about 2% in *V. orlovi* venom and less that 1% in all the others. Phospholipase B (PLB) was found in all venoms (less than 1%) and very low amount of Hya (0.01%) was detected in three venoms. Among the non-enzymatic proteins, Dis (13%) in the *V. renardi* venom, CTL (12%) in *V. kaznakovi*, CRISPs (12%) in *V. orlovi* and vascular endothelial growth factors (VEGF, 8%) in *V. nikolskii* were the most abundant ones in the venoms studied. The total amount of non-enzymatic proteins was about 13% in the *V. nikolskii* venom and about 27%–28% in all the others. In addition to the proteins mentioned above, nerve growth factor (NGF) and Kunitz were present in all the venoms (less than 1%) with exception of *V. kaznakovi*, where Kunitz was absent.

**Figure 3:**
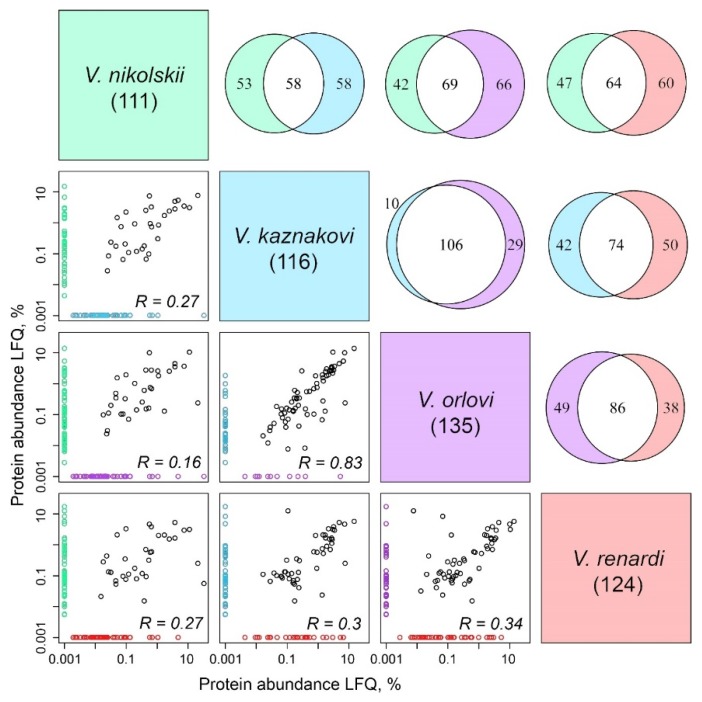
Protein number and abundance distributions for four *Vipera* species. ( The panels under the diagonal showing the species names) Individual protein abundance label-free quantification (LFQ) pairwise comparison. Proteins unique for a single species in a pair are highlighted in the corresponding color and for better visualization in logarithmic scale are assigned 0.001% abundance instead of real 0%. (The diagrams above the diagonal showing the species names) Pairwise Venn diagrams showing the number of common and unique proteins for each pair of the venoms.

Interestingly, comparison of both the nature of the identified proteins and their abundance showed very close venom compositions for the species *V. kaznakovii* and *V. orlovii.* The Pearson correlation coefficient for individual protein abundance LFQ was 0.83, while for the rest of pairs the correlation coefficient varied from 0.16 to 0.34 (Figure 3).

### 2.3. Identification of Endogenous Peptides in the Venoms

**Figure 4:**
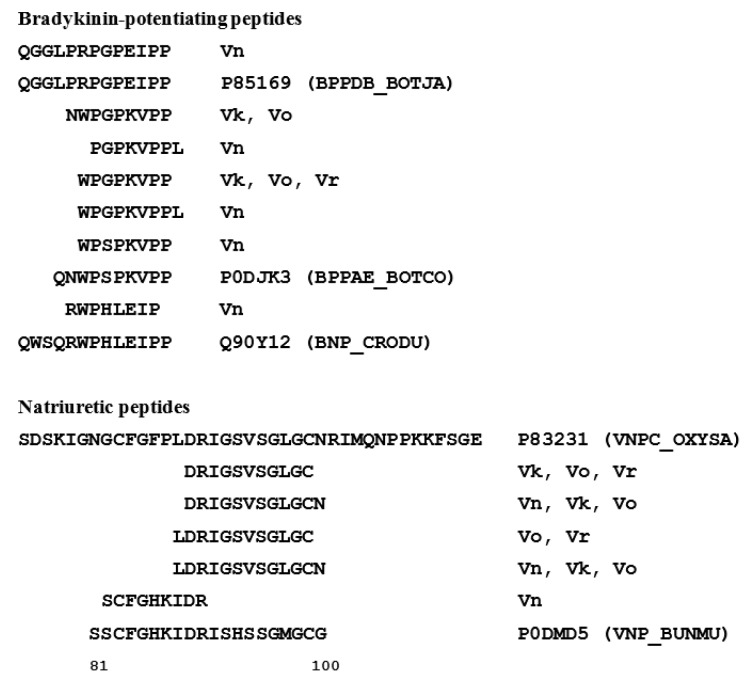
Bradykinin-potentiating and natriuretic peptides found in four viper venoms. P85169 (BPPDB_BOTJA)—Bradykinin-potentiating peptide 13b from *Bothrops jararaca*, P0DJK3 (BPPAE_BOTCO)—Bradykinin-potentiating peptide 10e from *Bothrops cotiara*, Q90Y12 (BNP_CRODU)—Bradykinin potentiating and C-type natriuretic peptides from *Crotalus durissus terrificus*, P83231 (VNPC_OXYSA)—Natriuretic peptide TNP-c from *Oxyuranus scutellatus canni* (Papuan taipan), and P0DMD5 (VNP_BUNMU)—amino acid sequence fragment 81–100 of Natriuretic peptide BM026 from *Bungarus multicinctus*. Vn, Vk, Vo, and Vr indicate *V. nikolskii*, *V. kaznakovi*, *V. orlovi*, and *V. renardi*, respectively.

It is well known that snake venoms may contain various peptides: several peptide families including bradykinin-potentiating peptides, natriuretic peptides, sarafotoxin, *etc.* were identified [17]. Moreover, the venoms studied in this work contain proteases, therefore their proteins may undergo proteolysis leading to generation of peptides. To study endogenously generated peptides in the venoms of interest, high molecular weight (MW) proteins were separated by ultrafiltration (10 KDa cut-off). The peptide fractions obtained were analyzed by LC-MS/MS in the same fashion as proteins, but without preliminary proteolysis. Peptides were searched at first against a full NCBI Serpentes database by Mascot search engine with 10% protein FDR (False Discovery Rate). A fusion database containing the peptidogenic proteins from Mascot search and the SwissProt Serpentes database was used for the final search in MaxQuant. Full NCBI database search with unspecific digestion failed in MaxQuant due to internal software limitations. In summary, 512 endogenous peptides from 80 proteins belonging to 13 protein families were found (Table S3). As expected, most of the peptides (462 peptides) belonged to proteins (48 proteins) which were earlier found in proteome, thus most likely representing venom protein degradation *in vivo* as a result of proteases and peptidases activity. At the same time, 50 peptides (Table S4) belonged to 32 unique proteins from nine protein families (Table 3). Among these, proteins in six families mostly had one peptide per protein, which can explain their identification only in the peptidome analysis as a result of a very low concentration of original proteins before degradation, so they were missed in the shotgun MS/MS selection in proteome analysis (or were beyond the taken FDR cut off). The largest number of unique peptides was found in proteins belonging to Dis and SVMP/Dis families: 25 peptides from 14 proteins were found. The peptides identified were mainly fragments of larger venom proteins. However, we found 12 peptides from proteins belonging to B-NAP family (Figure 4). These peptides may represent real endogenous peptides and possess their own biological activity. This is the first indication for the presence of bradykinin-potentiating and natriuretic peptides in venoms of vipers from the *Pelias* group.

## 3. Discussion

We have analyzed venom proteomes and peptidomes for four species of *Vipera*, for which there is no genomic or transcriptomic data published. For each species, the venoms of at least 15 individual animals were pooled for the analysis. Protein identification for such “non-sequenced” species is problematic for inherently database oriented bottom-up LC-MS/MS-based proteomics [18]. A possible solution is to use the protein sequences of closely related species, based on the assumption of their high homology level [18]. Thus, when the exact protein sequence is missing in the database, the protein might still be identified by partial/full homology with a known protein of another species. Here, we searched LC-MS/MS data against the database containing all the proteins from the taxon Serpentes in the NCBI database on the date of the experiment (the results are given in Table 1).

In the bottom-up proteomics, there are two major approaches for the quantitative analysis: (a) relative quantification of a single protein across samples; and (b) comparison of different proteins within a single sample. Principle (a) is based on the measurement of all the peptides belonging to a protein in several samples (and pair-wise peptide Fold Changes estimation) followed by protein Fold Change calculation as, e.g., mean or median value of the peptide fold changes. Principle (b) is based on the assumption that the sum of peptide peak areas (either all or just some of them, like in the top 3 theory [19,20]) for a given protein is proportional to its absolute abundance. Thus, comparison of these sums for two proteins is supposed to give the difference in their content within one sample. What is the most important, when making a comparison between several samples, the two approaches (a) and (b) are supposed to give consistent results.

In case of protein analysis of “non-sequenced” species, both these approaches encounter significant albeit different problems arising from the incomplete peptide identification due to the lack of adequate protein amino acid sequences in the search database. Principle (a) works best when as many as possible shared peptides per protein are identified and quantified for a pair of samples, since individual peptide measurements are prone to err due to possible post-translational modifications or isoforms. When it comes to different species, the number of shared peptides between samples goes down just because of different protein sequences. Besides, this approach works only when there are shared peptide sequences identified and quantified in both samples (recommended number of shared peptides for a reliable quantitation is 2). Thus, if a protein is unique for a sample, it cannot be quantified this way at all. Besides, it provides no data for concentration comparison between different proteins within a single sample.

Principle (b) was developed and verified for systems (artificial protein mixtures) where all the best flyer peptides for a protein (the peptides which have the best proportion between peptide concentration and intensity and thus have the maximum impact on the summed protein intensity) can be easily identified and quantified [19]. For “non-sequenced” species it would mess the final results through protein abundance underestimation if the missed peptides were among the best flyers for the given protein of some particular species but were overlooked because their amino acid sequence was missing in the database. For that, peptide MS/MS sequencing *de novo* might help a bit, but many peptides would still be missed for the reasons that are not clarified.

Here, we used two approaches to quantify proteins. First, we used MaxLFQ approach [21] which is basically principle (a), but it also uses absolute peptide intensities in addition to peptide FC comparison between samples (such results are labeled LFQ in Table 2). Second, we used direct comparison of sums of peptide intensities to make quantitation within each sample (such results are labeled INT in Table 2). The results of protein quantitation made by different methods gave quite similar results (Table 2, Table S1), especially when potential errors in individual protein contents were compensated by consolidation of proteins into families.

There is also a question of which types of peptides should be used for protein quantitation. Protein identification process deals not with separate proteins, but with protein groups, which are sets of individual proteins (at least partially homologous) sharing a set of identified peptide sequences. In the absence of unique specific peptides, no distinction between these proteins within a group can be made. A standard approach is to take as a hit the protein from a protein group which has a maximum number of assigned identified peptides. Thus, there are three types of peptides for a single protein group in the identification list: unique, razor and other (shared) peptides (MaxQuant terminology) [22,23]. Usually, protein groups have some unique peptides to pinpoint them as “correct” hits, but it is also possible that the number of unique peptides for a protein is zero. Absence of unique peptides means that all the peptides from the current protein group are shared and can be just as successfully assigned to some other protein groups. In such situation, the final set of protein groups shown in the identification list is the minimal one sufficient to explain all the identified peptides (Occam’s razor principle). Shared peptides are named “razor” when they belong to the protein group with the maximum total number of peptides among other possible protein groups. These razor peptides are used for quantitation (along with unique peptides), both LFQ and intensity based [24]. A shared peptide, which is “razor” for some particular group, is counted in “all peptides” in all the protein groups to which it can be potentially assigned, and “all peptides” list is used to calculate Sequence Coverage.

Importantly, any analytical method may prove only that the amount of the compound under investigation is below the method sensitivity, rather than show the absolute absence of the compound in the sample. This is specifically applicable for the LC-MS/MS-based shotgun identification principle which selects peptide ions pseudo-randomly, sometimes missing the peptides with very low intensities just because of a wrong choice. Thus, quantitation is much more reliable for showing the absence of the compound (or, more accurate, the concentration being lower than its Low Limit of Detection). MaxQuant features chromatogram alignment and the possibility to quantify peptides on the basis of similarity of their retention time and *m*/*z* in the sample, where they were identified by MS/MS and in another sample where this particular *m*/*z* signal got lost during the shot-gun selection for the MS/MS analysis (proteins with such peptides are marked “By matching” in “Identity Type” column in Tables S1 and S2). In this work the protein is considered to be identified (and considered as present) in the sample only if it has an MS/MS spectrum identified in this particular sample. However, for quantitation both MS/MS identified peptides and the peptides identified on the basis of the above described similarity were used. This might lead to apparent contradictions when there are no proteins identified, but the protein abundance is non-zero (like natriuretic peptides (B-NAP) in the *V. nikolskii* venom—0.01/0.01 (0) in Table 2).

At the present time, the genus *Vipera* includes 22 species, however it is not homogenous. Molecular phylogeny studies showed that this genus comprises the *V. aspis* group, the *V. ammodytes* complex, and the *Pelias* group as separate clades [25]. Of these clades, only snakes from the *Pelias* group inhabits Russia. The *Pelias* was further classified into two subgroups, one comprising *V. dinniki*, *V. kasnakovi*, and *V. ursinii*, and another including *V. berus*, *V. barani*, *V. nikolskii*, and *V. seoanei* [25]. The first subgroup was further subdivided into the “kaznakovi” complex, including *V. kasnakovi*, *V. orlovi* and some other closely related species, and the “ursinii” complex, in which *V. renardi* was included [26,27]. Earlier, for the vipers of the *Pelias* group, we have studied the venom toxicity towards crickets *Gryllus assimilis* [12] and found that it differed depending on feeding preferences. The snakes from the *V. renardi*, *V. lotievi*, *V. kaznakovi*, and *V. orlovi* species feed on a wide range of animals including insects, whereas the snakes from *V. berus* and *V. nikolskii* species do not include insects in their diet. The venom from vipers which hunt insects was found to possess a greater toxicity towards crickets. This suggests that the venom composition may greatly differ among these species. As concerns the toxicity to other animals, it was shown that the venom of *V. nikolskii* was more toxic than that of *V. berus* to frogs (9–11 µg/g *vs.* 30–52 µg/g) and mice (0.93 *vs* 1.58 µg/g) at intraperitoneal injection [28]. The venom of *V. renardi* was less toxic to mice (2.96 µg/g) than that of *V. berus* [28]. We were not able to find any data about toxicity of *V. orlovi* and *V. kaznakovi* venoms.

Regarding the danger to humans, the data about bites by these snakes are sparse. Most of the documented cases refer to steppe viper *V. renardi* and report that it usually has calm and timid behavior, is reluctant to bite, and seeks to escape. This viper bites only when it is in danger, for example, if the snake is suddenly stepped on or picked up. *V. renardi* is considered less dangerous to humans than common adder. The human fatalities as a consequence of steppe viper bites are not reliably known [29], though there are some cases of the death of horses and small ruminants. A picture of human envenomation is characterized mainly by local signs which include severe pain at the site of the bite, redness, swelling that spreads far beyond the site of the biting. In severe cases, drowsiness, dizziness, nausea, increase of heart rate, and reduction in body temperature may be observed [30].

Records of the bites of humans by the Caucasian viper *V. kaznakovi* and Nikolsky’s viper *V. nikolskii* are practically absent. However, *V. kaznakovi* may be dangerous. Solitary human deaths and livestock losses after Caucasian viper bites were mentioned [30]. We were able to find only one report about human fatalities after the Nikolsky’s viper bites [31]. No information on the *V. orlovi* bites is available.

It should be noted that the venoms of not all *Pelias* species were studied equally well. The venom of *V. berus* is the best characterized. As mentioned earlier, the *V. berus* venom displayed *in vitro* proteolytic, fibrinolytic, anticoagulant, and phospholipolytic activities. In mice, significant local tissue-damaging effects, including edema, hemorrhage and myonecrosis were observed for this venom [3]. Several proteins involved in manifestation of those effects were isolated from *V. berus* venom. These proteins included basic PLA2 [4], SVMP [5], LAAO [6] and some others.

The *V. nikolskii* species is phylogenetically very close to *V. berus* and is included in the same subgroup within the *Pelias* group. It is regarded as a *V. berus* subspecies in some publications. However, the analysis of the *V. nikolskii* venom has shown it to differ greatly from that of *V. berus.* Thus, two heterodimeric PLA2s were isolated from the *V. nikolskii* venom [10], but similar proteins are absent in *V. berus*. The data obtained in the present work are in good agreement with the published results; the basic and acidic PLA2 subunits forming heterodimeric enzymes account for more than 50% of the *V. nikolskii* venom (Table 1). Earlier, cDNA encoding SP nikobin and Kunitz type inhibitor in the *V. nikolskii* venom gland was cloned and sequenced [11]. In this study we have found that nikobin is the main SP in the *V. nikolskii* venom (more than 12% of the total protein content, Table 1) and Kunitz-type serine protease inhibitor ki-VN was also the main protein of the Kunitz family in this venom (about 0.6%, Table 1). CRISP, the sequence of which was also deduced from cDNA analysis [32], was found in the venom in fairly low amount (0.66%, Figure 2). Interestingly, the content of CRISPs was much higher in other venoms studied and accounted for 8%, 10% and 12% in *V. renardi*, *V. kaznakovi* and *V. orlovi* venoms, respectively (Figure 2).

The steppe viper *V. renardi* is included in the “ursinii” complex [33] while the other two vipers, *V. kaznakovi* and *V. orlovi*, belong to the “kaznakovi” complex. Among these vipers only the composition of the *V. renardi* venom was in some way studied [7]. The amino acid sequences for several PLA2s and Kunitz-type inhibitor were deduced from the cloned cDNA of venom gland. Some PLA2s and Kunitz protein were isolated from the venom. The most abundant was ammodytin I2d analogue. In this work we have found all the PLA2s described by Tsai *et al.* [7] in the *V. renardi* venom, Vur-PL2 having the highest content (Table 1). Interestingly, this viper venom has very high content of disintegrins which accounts for about 13% of total protein, while the *V. kaznakovi* and *V. orlovi* venoms contain less than 1% and in the *V. nikolskii* venom no disintegrins were detected.

There are no published data on the composition of the *V. kaznakovi* and *V. orlovi* venoms and they are characterized for the first time in this work. These two venoms have the highest similarity among the four ones studied (Figure 3) that confirms the inclusion of *V. orlovi* in the “kaznakovi” complex. They have a fairly high content of SVMPs (15%–16%), CTL (11%–12%) and CRISPs (11%–12%) (Figure 3). The *V. orlovi* venom has the highest amount of SP (24%) among the four venoms studied (Figure 3) and only *V. kaznakovi* contains a small quantity of hyaluronidase (Hya) at the level of 0.01%. However no Kunitz type proteins were detected in the latter venom.

Although a limited number of B-NAP proteins (one in *V. kaznakovi* and one in *V. orlovi*, Table 2) were detected in the proteome analysis, several peptides derived from proteins of this family were found in the peptidomes of all the venoms studied (Figure 4). The mature bradykinin-potentiating peptide QGGLPRPGPEIPP was observed in the *V. nikolskii* venom and several fragments of similar peptides were detected in the other analyzed venoms. Several fragments of C-type natriuretic peptides were found in all four venoms as well (Figure 4). It should be noted that no bradykinin-potentiating and C-type natriuretic peptides from the vipers of *Pelias* group were reported so far.

In total, 210 proteins (Table 1) and 512 endogenous peptides (Table S3) were identified in four viper venoms. The overwhelming majority of the proteins (98%–99% of the total protein content) and the peptides represented 14 snake venom protein families (Table 2). The comparison of our results with those for other snakes of the *Vipera* genus shows higher representation of venom protein families in our data (Table 4). For example, while Nuc and PLB were found in all venoms studied in this work, no proteins of these families were reported for other venoms from the *Vipera* species (Table 4).

Hya was observed in three of the studied venoms and this is also the first indication for the presence of this enzyme in the venoms of the *Vipera* species. We have found that the main components of all venom studied are PLA2s, while SVMPs were prevailing in venoms of *V.* *anatolica* [13] and *V. raddei* [14].

## 4. Conclusions

In this work, quantitative proteomic and peptidomic characterization of venoms from four vipers inhabiting Russia was done; the compositions of the venoms from *V. kaznakovi* and *V. orlovi*, which showed the highest similarity among the four studied species, were analyzed for the first time.

More than 200 proteins and over 500 peptides were detected in total in all four venoms. They represented 14 snake venom protein families. In all venoms studied, over 70% of the total proteins were enzymes, the highest enzyme content (85.7%) being in the *V. nikolskii* venom. The main components of the venoms were PLA2s, which accounted for 65% of total protein content in the *V. nikolskii* venom. For the first time, bradykinin-potentiating and C-type natriuretic peptides were reported for vipers of the *Pelias* group. Nucleic acid degrading enzymes and phospholipase B were found in the venoms of *Vipera* species for the first time.

Due to the low toxicity of the steppe viper, or a limited habitat of the Caucasian and Orlov’s vipers, these snakes do not pose an epidemiological threat to Russian population. However, the envenomation by Nikolsky’s viper, the venom of which was shown in this study to contain a considerable amount of neurotoxic phospholipase A_2_, may represent certain danger. An antiserum “Antigadyuka” (“Antiviper”) produced by Russian company “Allergen” is based on the venom of the common viper and may not be effective against the Nikolsky’s viper bites due to strong differences in the composition of the venoms. The need to consider the differences in the composition of the venoms in the antivenom production is discussed in recent publications [28,35] and should be taken into account by antiserum manufacturers.

## 5. Materials and Methods

The venoms of *V. kaznakovi*, *V. nikolskii*, *V. orlovi* and *V. renardi* vipers were obtained as described earlier [12]. The venoms from several individual animals were pooled as described in [12]. Snakes were captured in their natural habitat: *V. kaznakovi* in Krasnodar Territory near Adler, *V. nikolskii* in Penza region near Zubrilovo village, *V. orlovi* in Krasnodar Territory at Mikhaylovskiy mountain pass and *V. renardi* in Krasnodar Territory near Beysugskiy firth.

### 5.1. In-Solution Trypsin Digestion of Venom Samples

Lyophilized venom sample (100 μg each) was dissolved in 10 μL of a buffer containing 100 mM ammonium bicarbonate (ABC), 5% sodium deoxycholate (SDC) and 5 mM dithiothreitol (DTT) and incubated for 40 min at 60 °C to reduce cysteine residues. Then, 5 μL of 50 mM iodoacetamide (IAA) water solution was added and the mixture was incubated 30 min at RT, in the dark. Residual IAA was neutralized by 5 μL of 50 mM DTT and sample was diluted with 50 μL 50 mM ABC and trypsin was added in a 1:100 (enzyme/protein) ratio to the final volume 100 μL and the protein concentration ~1 mg/mL. Samples were incubated overnight at 37 °C. Trypsin was deactivated by addition of 5 μL of 10% TFA. Tryptic peptides were desalted using reverse-phase solid extraction cartridges Discovery DSC-18 (100 mg) (Supelco, Bellefonte, PA, USA) according to the manufacturer protocol. Final peptide solution was dried in vacuum and stored at −80 °C prior to LC-MS/MS analysis.

### 5.2. Endogenous Venom Peptides Isolation

Endogenous peptides from venom samples were isolated using C_18_ StageTips [36]. To make StageTips, two pieces of C18 Empore extraction disk were cut using blunt-ended 16-gauge needle and packed into a P200 pipette tip. Membranes were conditioned by 20 μL of methanol and equilibrated by 20 μL of 0.1% aqua TFA. Venom solutions were applied onto the conditioned tips, followed by membrane washing with 20 μL of 0.1% aqua TFA. Peptides were eluted by 20 μL of 80% ACN, 0.1% TFA. Eluates were dried in vacuum and stored at −80 °C prior to LC-MS/MS analysis.

### 5.3. LC-MS/MS Analysis

Analysis was performed on the QExactive HF mass-spectrometer (Thermo Scientific, Waltham, MA, USA) coupled to the Dionex 3000 RSLCnano HPLC system (Thermo Scientific, Waltham, MA, USA). The HPLC system was configured in a trap-elute mode. An analytical column (75 μm × 150 mm) and a precolumn (100 μm × 10 mm) were in-house packed with Aeris Peptide C18 2.6 μm sorbent (Phenomenex, Torrance, CA, USA). Samples were loaded on the precolumn for 10 min at 3 mL/min with buffer A (3% AcN, 96.9% H2O, 0.1% FA), followed by separation at 300 nL/min with the 4%–55% gradient of buffer B (80% AcN, 19.9% H_2_O, 0.1% FA).

Mass-spectrometer experiment consisted of one full survey MS1 scan followed by 20 dependent MS2 scans for the most intense ions. MS1 spectra were acquired in the profile mode in mass range 350–1400 *m*/*z*, maximum IT time 100 ms, AGC target 3e6, resolution 60000. Dependent MS2 scan were performed at resolution 15000 for 200–2000 *m*/*z* mass range, AGC target 1e5, maximum IT 25 ms, isolation window 1.4 *m*/*z*. Dynamic exclusion was set to 30 s.

### 5.4. LC-MS/MS Data Analysis

Data analysis was performed in the MaxQuant software (V. 1.5.3.30, Max Planck Institute of Biochemistry, Martinsried, Germany, 2016). Proteomic LC-MS/MS data was searched with the Andromeda search engine incorporated in the MaxQuant software against NCBI Serpentes DataBase exported from the NCBI web-site [37] for the Taxon Serpentes 2015/11/17 and containing 134677 entries with the following parameters: digestion Trypsin/P; max number of miscleavages 2; include contaminants; fixed modification: carbamidomethyl (Cys); variable modifications: Oxidation (Met), Acetylation (N-term), Deamidation (Asn, Gln); min peptide length 6; max peptide MW 5500; PSM FDR 0.01; protein FDR 0.05; decoy mode: revert; min number of peptides for identification 1; razor protein FDR; second peptide; match between runs; LFQ quantitation with minimum 2 peptide pairs; and stabilize large LFQ ratios. Full set of MaxQuant parameters for the analysis can be found in the Supplementary Data file mqpar_proteins.xml.

Peptidomic LC-MS/MS data were searched with the Mascot search engine against the same full NCBI Serpentes data base with the following parameters: MS tolerance 5 ppm; MS/MS tolerance 0.01 Da; charge: +1, +2, +3; fixed modification: carabamidomethyl (Cys); variable modifications: Oxidation (Met), Deamidation (Asn, Gln); enzyme none. Mascot results were reprocessed in the Scaffold software and identified peptidogenic proteins (protein FDR 10%) for all four venoms were added to the SwissProt Serpentes database exported from the NCBI web-site on 2015/11/30 and contains 2567 sequences to generate a fused database. This database was used for Andromeda search in MaxQuant software with the digestion parameter set to unspecific. Peptide length for unspecific digestion search was from 6 to 50 amino acids. The rest of the parameters were the same as for proteome data analysis, however in the peptidogenic protein features in the in the “Number of Unique and Razor Peptides (NoURP)” column, the number of peptides corresponds to that before PEP-based filtration.

Results were processed in the Perseus (V. 1.5.2.6, Max Planck Institute of Biochemistry, Martinsried, Germany, 2016) and Excel software (V. 12.06743.5000, Microsoft Corporation, Redmond, WA, USA, 2007) and with the use of R.

## Figures and Tables

**Table 1:** List of proteins identified in Russian viper venoms.

Protein No. in SuppData	Protein Name	Taxon	Protein Family ^1^	MW, KDa	Seq Cov, %	*V. nikolskii*		*V. kaznakovi*		*V. orlovi*		*V. renardi*	
						Prot Abun INT, %	Seq Cov, %	Prot Abun INT, %	Seq Cov, %	Prot Abun INT, %	Seq Cov, %	Prot Abun INT, %	Seq Cov, %
9	Natriuretic peptide	*Pseudonaja textilis*	B-NAP	14	9.8	0.007	9.8	0.029	9.8	0.012	9.8	-	-
2	Hemoglobin subunit alpha	*Vipera aspis*	BP	15	6.4	0	6.4	-	-	-	-	0	6.4
3	Hemoglobin subunit beta-2	*Naja naja*	BP	16	7.5	0.016	7.5	-	-	-	-	-	-
4	Hemoglobin subunit beta	*Erythrolamprus miliaris*	BP	15	23.3	0.018	19.2	-	-	-	-	0	11.6
33	Alpha globin	*Hydrophis melanocephalus*	BP	16	19.7	0.011	9.2	0	9.2	-	-	0.010	19.7
34	Alpha globin	*Elaphe climacophora*	BP	11	25.2	-	-	-	-	-	-	0.032	25.2
116	Murinoglobulin-2	*Ophiophagus hannah*	BP	143	2.7	-	-	-	-	0.008	1.8	0.002	2.2
124	Serum albumin	*Protobothrops flavoviridis*	BP	69	5.4	0.013	5.4	-	-	0.002	5.2	-	-
158	Hemoglobin subunit alpha	*Hydrophis gracilis*	BP	15	15.6	0.013	15.6	0.002	15.6	-	-	0.017	15.6
165	Hemoglobin subunit alpha	*Crotalus horridus*	BP	15	11.3	0.012	11.3	0.003	11.3	-	-	0.013	11.3
171	Hemoglobin subunit beta-1	*Boiga irregularis*	BP	16	21.8	0	21.8	-	-	-	-	0	21.8
177	Transferrin	*Crotalus adamanteus*	BP	77	3.1	0.006	3.1	-		0.004	3.1	0.009	3.1
199	Hemoglobin subunit beta-2	*Thamnophis sirtalis*	BP	16	24.5	0.021	18.4	-	-	-	-	0.020	12.9
200	Hemoglobin subunit beta-1	*Thamnophis sirtalis*	BP	16	19.7	0.002	19.7	-	-	-	-	-	-
206	Serum albumin-like	*Thamnophis sirtalis*	BP	57	8.0	0.012	8.0	-	-	-	-	-	-
180	Cathepsin B-like protein	*Crotalus adamanteus*	CP	37	5.0	0	5.0	-	-	-	-	-	-
7	Cysteine-rich venom protein	*Philodryas patagoniensis*	CRISP	1	64.3	-	-	0.037	64.3	0.002	64.3	-	-
18	Cysteine-rich seceretory protein Dr-CRPK	*Daboia russelii*	CRISP	26	24.7	-	-	0.170	24.7	0.107	21.8	-	-
19	Cysteine-rich seceretory protein Dr-CRPB	*Daboia russelii*	CRISP	25	17.6	-	-	0.033	17.6	0.006	15.8	0	17.6
89	Cysteine-rich venom protein	*Protobothrops jerdonii*	CRISP	26	22.5	-	-	0.148	22.5	0.149	22.5	-	-
90	Cysteine-rich venom protein	*Vipera nikolskii*	CRISP	24	83.3	-	-	0.112	77.4	0.050	80.1	0.116	68.3
91	Cysteine-rich venom protein	*Vipera berus*	CRISP	26	77	0.345	74.1	7.916	71.5	9.924	74.1	5.125	63.2
145	Cysteine-rich venom protein triflin	*Protobothrops flavoviridis*	CRISP	24	23.1	0.070	23.1	2.474	23.1	2.065	23.1	3.016	23.1
22	Snaclec 1	*Sistrurus catenatus edwardsii*	CTL	17	6.2	-	-	1.404	6.2	0.168	6.2	-	-
23	C-type lectin lectoxin-Thr1	*Thrasops jacksonii*	CTL	18	7.0	0.010	6.3	0.060	7.0	0.110	7	0.069	7
24	Snaclec A13	*Macrovipera lebetina*	CTL	15	33.6	-	-	2.042	29.0	2.555	33.6	-	-
25	Snaclec A15	*Macrovipera lebetina*	CTL	17	46.8	0.710	46.8	0.867	46.8	0.783	46.8	0.524	46.8
26	Snaclec B7	*Macrovipera lebetina*	CTL	15	27.2	-	-	3.475	27.2	1.274	27.2	-	-
101	Snaclec VP12 subunit A	*Daboia palaestinae*	CTL	12	26.2	-	-	0.008	26.2	-	-	-	-
102	Snaclec VP12 subunit B	*Daboia palaestinae*	CTL	15	25.6	-	-	0.084	25.6	0.029	17.6	-	-
153	C-type lectin J	*Echis coloratus*	CTL	18	14.6	0.589	14.6	0.740	14.6	0.579	14.6	0.477	14.6
154	C-type lectin H	*Echis coloratus*	CTL	18	10.8	0.050	10.1	0.935	10.8	0.909	10.8	0.172	10.8
155	C-type lectin E	*Echis coloratus*	CTL	11	12.1	0.011	7.1	-	-	0.008	7.1	0.045	12.1
156	C-type lectin B	*Echis coloratus*	CTL	13	7.1	-	-	0.048	7.1	0.005	7.1	-	-
157	C-type lectin A	*Echis coloratus*	CTL	18	10.1	0.014	10.1	-	-	-	-	-	-
159	Snaclec coagulation factor X-activating enzyme light chain 2	*Macrovipera lebetina*	CTL	18	6.3	0.039	6.3	0.199	6.3	0.340	6.3	0.051	6.3
173	C-type lectin-like protein 3B	*Macrovipera lebetina*	CTL	17	42.6	2.457	42.6	2.758	42.6	2.500	42.6	1.545	42.6
174	C-type lectin-like protein 4B	*Macrovipera lebetina*	CTL	17	14.7	0.018	12.0	-	-	0.017	12.0	0.353	14.7
175	Snaclec dabocetin subunit alpha	*Daboia siamensis*	CTL	17	6.5	-	-	0.443	6.5	0.064	6.5	-	-
181	Snaclec tokaracetin subunit beta	*Protobothrops tokarensis*	CTL	4	32.5	-	-	0.026	32.5	-	-	-	-
210	Snaclec anticoagulant protein subunit B	*Deinagkistrodon acutus*	CTL	14	12.2	-	-	0.352	12.2	0.124	12.2	-	-
14	Disintegrin VB7A	*Vipera berus berus*	Dis	7	76.6	-	-	0.111	23.4	0.008	23.4	12.932	76.6
15	Disintegrin VB7B	*Vipera berus berus*	Dis	6	70.3	-	-	0.008	29.7	-	-	0.935	70.3
16	Disintegrin VLO4	*Macrovipera lebetina obtusa*	Dis	7	38.5	-	-	0.011	24.6	0.006	24.6	0.034	38.5
17	Disintegrin VA6	*Vipera ammodytes ammodytes*	Dis	7	23.4	-	-	-	-	-	-	0.133	23.4
191	Disintegrin lebein-1-alpha	*Macrovipera lebetina*	Dis	12	30.6	-	-	0.389	9.0	0.578	9.0	0.006	21.6
86	Hyaluronidase	*Echis ocellatus*	Hya	52	13.6	0.007	13.6	0.011	7.3	0.004	2.7	-	-
176	Hyaluronidase	*Crotalus adamanteus*	Hya	52	6.7	0.004	6.7	0.007	6.7	0.003	2.0	-	-
27	Inhibitor, chymotrypsin	*Vipera ammodytes*	Kunitz	7	40.0	0.002	24.6	-	-	0.047	29.2	0.478	29.2
80	Protease inhibitor 3	*Walterinnesia aegyptia*	Kunitz	8	21.0	-	-	-	-	0	21.0	0.005	21.0
81	Kunitz-type serine protease inhibitor Vur-KIn	*Vipera renardi*	Kunitz	7	39.4	0.015	39.4	-	-	0.074	39.4	0.241	39.4
87	KP-Sut-1	*Suta fasciata*	Kunitz	13	9.4	0.072	9.4	-	-	-	-	0.077	9.4
142	Kunitz-type serine protease inhibitor ki-VN	*Vipera nikolskii*	Kunitz	10	34.7	0.610	34.7	-	-	-	-	-	-
5	l-amino-acid oxidase	*Macrovipera lebetina*	LAAO	12	42.1	0.041	41.1	1.388	42.1	1.631	41.1	1.786	41.1
65	l-amino-acid oxidase	*Echis ocellatus*	LAAO	56	6.0	-	-	-	-	0	6.0	-	-
66	l-amino-acid oxidase	*Vipera ammodytes ammodytes*	LAAO	54	13.2	-	-	0.009	11.2	0.012	11.2	0.007	13.0
75	l-amino-acid oxidase	*Daboia russelii*	LAAO	56	11.7	0	5.8	0.316	7.7	0.045	11.7	0.049	9.9
92	Kunitz-type serine protease inhibitor PIVL	*Macrovipera lebetina transmediterranea*	LAAO	10	8.4	-	-	-	-	0	8.4	0.021	8.4
94	l-amino-acid oxidase	*Gloydius halys*	LAAO	55	9.9	-	-	-	-	0	7.4	-	-
107	l-amino acid oxidase	*Ovophis okinavensis*	LAAO	58	11.0	0.001	3.9	1.893	6.8	2.106	6.8	1.378	10.9
144	l-amino acid oxidase	*Protobothrops elegans*	LAAO	57	5.7	-	-	0	5.7	-	-	-	-
152	l-amino acid oxidase B variant 1	*Echis coloratus*	LAAO	56	17.1	-	-	0.425	13.1	0.213	12.9	0.216	13.9
164	l-amino-acid oxidase	*Crotalus horridus*	LAAO	58	11.2	0.001	4.5	0.042	6.6	0.005	5.8	0	5.8
183	l-amino-acid oxidase	*Bothrops moojeni*	LAAO	54	12.1	0.022	6.5	0.256	7.1	0.178	9.4	0.098	9.2
62	Venom nerve growth factor 2	*Daboia russelii*	NGF	27	14.4	-	-	0	14.4	-	-	-	-
63	Venom nerve growth factor	*Vipera ursinii*	NGF	27	25.5	0.285	18.1	0.122	18.1	0.254	25.5	0.093	18.1
76	Snake venom 5′-nucleotidase	*Gloydius blomhoffii blomhoffii*	Nuc	6	27.8	0.013	27.8	-	-	-	-	-	-
110	5′-nucleotidase	*Ovophis okinavensis*	Nuc	55	26.6	0.091	26.6	0.012	9.1	0.018	16.5	0.057	22.4
125	Phosphodiesterase	*Macrovipera lebetina*	Nuc	96	35.4	0.243	35.4	0.103	21.4	0.088	20.7	0.038	19.5
126	5′-nucleotidase	*Macrovipera lebetina*	Nuc	45	58.1	0.563	54.4	0.046	14.5	0.097	30.4	0.226	39.2
127	Venom phosphodiesterase 2	*Crotalus adamanteus*	Nuc	91	14.0	0.007	14.0	-	-	-	-	-	-
132	Ectonucleotide pyrophosphatase/phosphodiesterase family member 3-like	*Python bivittatus*	Nuc	93	7.5	0.004	7.5	0.002	3.5	0.003	3.5	-	-
170	Ectonucleotide pyrophosphatase/phosphodiesterase family member 3	*Boiga irregularis*	Nuc	100	4.5	-	-	0.441	3.3	1.521	2.6	-	-
73	Proactivator polypeptide-like	*Crotalus adamanteus*	OP	58	19.7	0.036	19.7	0.011	5.2	0.024	8.7	0.006	2.5
104	ArfGAP with SH3 domain ankyrin repeat and PH domain 3	*Micrurus fulvius*	OP	107	2.3	-	-	0.027	2.3	0.033	2.3	-	-
113	Uncharacterized protein	*Ophiophagus hannah*	OP	46	3.4	-	-	0.040	3.4	0.015	3.4	-	-
115	78 kDa glucose-regulated protein	*Ophiophagus hannah*	OP	67	6.4	0	2.8	0.004	4.4	0	2.0	-	-
117	WD repeat-containing protein 67	*Ophiophagus hannah*	OP	113	0.8	-	-	-	-	-	-	0.652	0.8
118	Pituitary adenylate cyclase-activating polypeptide type I receptor	*Ophiophagus hannah*	OP	7	18.2	0.008	18.2	-	-	-	-	-	-
119	Iron-responsive element-binding protein 2	*Ophiophagus hannah*	OP	90	1.8	-	-	0.536	1.8	0.482	1.8	0.224	1.8
122	Glutathione peroxidase	*Ophiophagus hannah*	OP	29	21.6	0.078	18.9	0.047	16.7	0.095	21.6	0.081	16.7
128	PiggyBac transposable element-derived protein 5	*Python bivittatus*	OP	69	2.7	-	-	-	-	-	-	0.067	2.7
129	Calmodulin-lysine N-methyltransferase	*Python bivittatus*	OP	14	12.3	-	-	-	-	0	12.3	-	-
130	Dipeptidase 2	*Python bivittatus*	OP	46	7.0	-	-	-	-	-	-	0	7.0
131	Serine/threonine-protein phosphatase 6 regulatory subunit 1 isoform X3	*Python bivittatus*	OP	92	1.9	-	-	0.199	1.9	0.117	1.8	0.279	1.8
133	E3 ubiquitin-protein ligase MARCH8-like isoform X8	*Python bivittatus*	OP	30	7.2	-	-	-	-	-	-	0.702	7.2
134	Nucleolar and coiled-body phosphoprotein 1 isoform X5	*Python bivittatus*	OP	104	1.4	-	-	-	-	0	1.4	-	-
135	E3 ubiquitin-protein ligase UBR4	*Python bivittatus*	OP	555	0.2	0	0.2	-	-	-	-	-	-
136	Extracellular matrix protein 1	*Python bivittatus*	OP	24	8.6	0.019	8.6	-	-	0.004	8.6	-	-
137	Receptor-type tyrosine-protein phosphatase gamma-like	*Python bivittatus*	OP	102	1.9	-	-	0.026	1.9	0.018	1.9	0.018	1.9
138	Nurim-like	*Python bivittatus*	OP	32	7.7	0.010	7.7	-	-	-	-	-	-
162	Dickkopf-related protein 3-like	*Crotalus horridus*	OP	31	5.0	-	-	0.005	5	0.007	5.0	-	-
168	RNA binding motif protein 6	*Boiga irregularis*	OP	59	3.5	0.007	3.5	-	-	-	-	-	-
172	Filamin-B isoform 15	*Boiga irregularis*	OP	282	0.9	-	-	0.010	0.9	-	-	-	-
197	Leucine-rich repeats and immunoglobulin-like domains protein 1	*Thamnophis sirtalis*	OP	48	1.4	0.010	1.4	0.028	1.4	0.026	1.4	-	-
201	Peroxiredoxin-4-like	*Thamnophis sirtalis*	OP	31	8.7	0.002	8.7	-	-	-	-	-	-
202	Obscurin	*Thamnophis sirtalis*	OP	1024	0.5	0.109	0.4	0.107	0.2	-	-	-	-
203	CCR4-NOT transcription complex subunit 3	*Callithrix jacchus*	OP	12	15.7	-	-	0.008	15.7	0.046	15.7	-	-
205	Tyrosine-protein phosphatase non-receptor type 20	*Thamnophis sirtalis*	OP	50	4.0	-	-	0.019	4.0	0.020	4.0	-	-
208	Protein BANP	*Thamnophis sirtalis*	OP	40	4.4	-	-	0.064	4.4	0.076	4.4	0.119	4.4
209	Microtubule-associated serine/threonine-protein kinase 1-like	*Thamnophis sirtalis*	OP	94	0.7	-	-	-	-	-	-	0	0.7
10	Basic phospholipase A_2_ chain HDP-1P	*Vipera nikolskii*	PLA2	13	86.9	20.289	86.9	5.460	9.0	0.230	9.0	0.288	27.0
13	Basic phospholipase A_2_ B chain	*Vipera aspis zinnikeri*	PLA2	13	80.3	0.130	80.3	-	-	-	-	-	-
28	Phospholipase A_2_ II	*Vipera aspis*	PLA2	5	40.4	0.018	19.2	-	-	0.009	21.2	-	-
29	Phospholipase A_2_ III	*Vipera aspis*	PLA2	5	66.0	0.002	66.0	6.579	66.0	5.099	66.0	0	66.0
30	Acidic phospholipase A_2_ PLA-1	*Eristicophis macmahoni*	PLA2	13	22.3	-	-	-	-	-	-	0.419	22.3
31	Acidic phospholipase A_2_ PLA-2	*Eristicophis macmahoni*	PLA2	13	29.8	0	28.9	-	-	0	21.5	0.388	29.8
32	Acidic phospholipase A_2_ homolog vipoxin A chain	*Vipera ammodytes meridionalis*	PLA2	13	94.3	34.017	94.3	-	-	-	-	0.048	18.9
56	Basic phospholipase A_2_ 3	*Daboia russelii*	PLA2	13	27.3	-	-	0.003	12.4	0.002	12.4	0.048	27.3
57	Phospholipase A2	*Agkistrodon piscivorus*	PLA2	13	27.6	0	16.3	-	-	-	-	-	-
58	Phospholipase A_2_ homolog P-elapitoxin-Aa1a beta chain	*Acanthophis antarcticus*	PLA2	3	22.6	-	-	0.165	22.6	0.127	22.6	0.030	22.6
67	Acidic phospholipase A_2_ RV-7	*Daboia siamensis*	PLA2	13	45.1	1.078	45.1	-	-	-	-	-	-
78	Basic phospholipase A_2_ Pla2Vb	*Vipera berus berus*	PLA2	15	46.4	-	-	0	13.8	0.056	46.4	-	-
79	Acidic phospholipase A_2_ Vur-PL3	*Vipera renardi*	PLA2	15	69.3	-	-	12.956	67.2	12.705	67.2	7.113	58.4
82	Acidic phospholipase A_2_ PL1	*Vipera renardi*	PLA2	15	75.4	4.057	70.3	5.097	59.4	4.080	52.9	10.603	75.4
83	Acidic phospholipase A_2_ Vur-PL2B	*Vipera renardi*	PLA2	15	72.3	-	-	0	19.7	0.041	19.7	14.999	72.3
84	Basic phospholipase A_2_ homolog Vur-S49	*Vipera renardi*	PLA2	15	63.0	-	-	0.009	18.8	0.055	18.8	7.676	63.0
85	Basic phospholipase A_2_ vurtoxin	*Vipera renardi*	PLA2	15	50.0	-	-	-	-	-	-	4.220	50.0
95	Ammodytin I1	*Vipera aspis aspis*	PLA2	15	56.5	0.032	56.5	0.048	56.5	0.040	44.9	0.026	39.9
96	Ammodytin I1	*Vipera ammodytes montandoni*	PLA2	15	75.4	1.389	75.4	1.428	75.4	0.524	63.8	1.710	59.4
97	Ammodytin I2	*Vipera aspis aspis*	PLA2	15	19.7	-	-	-	-	-	-	0	19.7
98	Ammodytin I2	*Vipera berus berus*	PLA2	15	36.5	-	-	0.021	31.4	-	-	-	-
99	Ammodytin I2	*Vipera ursinii*	PLA2	15	45.3	-	-	-	-	-	-	0.017	45.3
100	Ammodytin L	*Vipera ammodytes ammodytes*	PLA2	15	14.5	-	-	-	-	-	-	0.009	14.5
139	Basic phospholipase A_2_ vipoxin B chain	*Vipera ammodytes meridionalis*	PLA2	13	80.3	0.086	80.3	-	-	-	-	-	-
151	Phospholipase A_2_ Group IIE	*Echis coloratus*	PLA2	13	5.8	-	-	-	-	-	-	0.020	5.8
161	Basic phospholipase A_2_	*Azemiops feae*	PLA2	15	7.2	-	-	-	-	0.012	7.2	0.016	7.2
193	Phospholipase A_2_-III	*Daboia russelii*	PLA2	13	13.1	0.019	13.1	-	-	-	-	-	-
195	Phospholipase A_2_ ammodytin I1	*Vipera nikolskii*	PLA2	15	75.4	4.779	75.4	4.663	75.4	4.289	63.8	-	-
196	Basic phospholipase A_2_ chain HDP-2P	*Vipera nikolskii*	PLA2	15	69.6	0.068	69.6	-	-	-	-	0.006	31.2
109	Phospholipase b	*Ovophis okinavensis*	PLB	64	20.8	-	-	0.015	13.6	0.037	20.1	0.057	15.0
114	Phospholipase B-like 1	*Ophiophagus hannah*	PLB	58	16.8	0.022	7.0	0.085	12.0	0.104	16.2	0.088	10.2
169	Phospholipase B	*Boiga irregularis*	PLB	64	15.6	-	-	0.020	11.6	0.034	11.2	0.034	9.9
179	Phospholipase B	*Crotalus adamanteus*	PLB	64	26.9	0.080	16.5	0.218	15.9	0.321	21.0	0.334	12.7
6	Unassigned	*Calloselasma rhodostoma*	SP	24	9.4	-	-	0.007	5.5	0.041	8.1	0.006	8.1
11	Snake venom serine protease pallase	*Gloydius halys*	SP	26	13.1	0.002	9.3	0.005	10.6	0.002	11.9	0	10.6
12	Snake venom serine protease ussurase	*Gloydius ussuriensis*	SP	26	5.6	0.077	5.6	-	-	-	-	-	-
21	Thrombin-like enzyme KN-BJ 2	*Bothrops jararaca*	SP	27	11.3	1.017	11.3	0.329	11.3	1.944	11.3	0.050	11.3
35	Serine protease	*Echis coloratus*	SP	28	8.5	-	-	0.043	8.5	0.172	8.5	0.087	8.5
36	Serine protease	*Echis ocellatus*	SP	24	6.3	0.515	6.3	1.980	3.6	2.401	6.3	0.694	6.3
37	Serine protease	*Echis coloratus*	SP	25	7.3	-	-	0.079	4.7	0.047	7.3	-	-
38	Serine protease	*Echis coloratus*	SP	25	12.0	0.004	12.0	0	9.4	0.007	12.0	-	-
39	Serine protease	*Echis coloratus*	SP	26	5.9	0.110	5.9	0.048	3.4	0.015	5.9	0.005	5.9
40	Serine protease	*Echis carinatus sochureki*	SP	25	8.1	0.021	5.5	-	-	-	-	0.036	5.5
64	Factor V activator RVV-V gamma	*Daboia siamensis*	SP	25	13.7	0.006	11.1	0.473	8.1	0.075	5.6	-	-
69	Serine protease VLSP-3	*Macrovipera lebetina*	SP	28	15.5	3.526	15.5	2.548	12.0	3.479	14.3	1.303	14.3
70	Beta-fibrinogenase	*Macrovipera lebetina*	SP	28	18.3	0.017	18.3	0.008	9.3	0.078	11.7	0.224	11.7
71	Chymotrypsin-like protease VLCTLP	*Macrovipera lebetina*	SP	28	29.6	0.069	29.6	-	-	-	-	-	-
74	Snake venom serine protease nikobin	*Vipera nikolskii*	SP	28	43.2	12.595	43.2	2.334	34.2	8.515	32.3	1.732	28.0
77	Snake venom serine protease pallabin	*Gloydius halys*	SP	28	12.3	0.002	8.8	-	-	-	-	0.010	10.0
103	Kallikrein-CohID-1	*Crotalus oreganus helleri*	SP	28	10.8	-	-	-	-	-	-	0	10.8
105	Serine protease	*Protobothrops flavoviridis*	SP	18	17.4	0	17.4	-	-	-	-	-	-
106	Serine protease	*Ovophis okinavensis*	SP	10	23.3	0.061	20.0	0.028	23.3	0.352	20.0	0.067	23.3
140	Factor V activator	*Macrovipera lebetina*	SP	28	18.9	0.049	13.9	0.776	11.6	0.088	11.2	0.005	5.0
141	Venom serine proteinase-like protein 2	*Macrovipera lebetina*	SP	28	30.8	1.028	30.8	0.896	26.5	1.680	25.4	1.595	26.5
163	Serine proteinase 1	*Crotalus horridus*	SP	28	13.2	1.023	10.9	0.274	4.3	2.042	6.6	-	-
187	Snake venom serine protease HS112	*Bothrops jararaca*	SP	27	14.9	-	-	0.157	12.5	0.323	14.9		-
188	Snake venom serine protease KN6	*Trimeresurus stejnegeri*	SP	28	3.5	0.036	3.5	-	-	-	-	-	-
189	Snake venom serine protease 5	*Trimeresurus stejnegeri*	SP	28	11.6	0	11.6	-	-	-	-	-	-
190	Snake venom serine protease catroxase-2	*Crotalus atrox*	SP	27	17.4	0.352	17.4	0.162	8.5	0.531	10.9	0.012	10.9
198	Rho GTPase-activating protein 28-like	*Thamnophis sirtalis*	SP	24	3.2	-	-	0.811	3.2	0.822	3.2	0.207	3.2
41	Metalloproteinase	*Echis carinatus sochureki*	SVMP	69	14.6	-	-	1.488	8.7	1.023	11.5	0.401	14.6
42	Metalloproteinase	*Echis carinatus sochureki*	SVMP	68	6.9	-	-	-	-	-	-	0.107	6.9
43	Metalloproteinase	*Echis carinatus sochureki*	SVMP	27	6.5	-	-	2.987	6.5	2.973	6.5	1.419	6.5
44	Metalloproteinase	*Echis coloratus*	SVMP	56	6.3	-	-	-	-	-	-	0.004	6.3
45	Metalloproteinase	*Echis coloratus*	SVMP	56	11.2	-	-	0.109	9.4	0.080	9.4	0.116	9.0
46	Metalloproteinase	*Echis coloratus*	SVMP	66	10.7	-	-	0	9.2	0	9.2	0	10.7
47	Metalloproteinase	*Echis coloratus*	SVMP	69	3.1	-	-	-	-	-	-	0.008	3.1
48	Metalloproteinase	*Echis coloratus*	SVMP	69	7.4	-	-	0.208	7.4	0.435	4.7	0.058	4.9
49	Metalloproteinase	*Echis coloratus*	SVMP	68	8.8	-	-	-	-	-	-	0	8.8
50	Metalloproteinase	*Echis coloratus*	SVMP	68	13.8	-	-	0	8.9	0	5.2	0.488	4.9
51	Metalloproteinase	*Echis coloratus*	SVMP	61	5.4	-	-	2.479	5.4	2.296	5.4	1.071	5.4
52	Metalloproteinase	*Echis coloratus*	SVMP	68	6.4	-	-	0.019	6.4	0.012	5.6	0.021	6.4
53	Metalloproteinase	*Echis pyramidum leakeyi*	SVMP	62	8.1	0.009	2.5	-	-	-	-	-	-
54	Metalloproteinase	*Echis pyramidum leakeyi*	SVMP	46	5.4	-	-	0.019	5.4	0.014	5.4	-	-
55	Metalloproteinase	*Echis carinatus sochureki*	SVMP	54	5.6	-	-	-	-	-	-	0.010	5.6
59	Group III snake venom metalloproteinase	*Echis ocellatus*	SVMP	62	10.7	0	3.1	1.254	6.0	1.744	9.0	0.651	10.7
61	Snake venom metalloproteinase VMP1	*Agkistrodon piscivorus leucostoma*	SVMP	46	6.8	-	-	-	-	-	-	0.833	6.8
72	Snake venom metalloproteinase	*Crotalus adamanteus*	SVMP	68	9.0	-	-	0.005	3.4	0.016	4.7	0.085	7.7
93	H3 metalloproteinase 1	*Vipera ammodytes ammodytes*	SVMP	68	43.5	0.611	32.0	3.415	33.3	2.970	35.9	2.737	37.3
108	P-III metalloprotease	*Ovophis okinavensis*	SVMP	16	12.1	-	-	2.110	12.1	1.999	12.1	0.945	12.1
112	Metalloproteinase H4-A	*Vipera ammodytes ammodytes*	SVMP	68	14.2	0.022	11.4	0.342	4.2	0.006	4.2	-	-
143	Snake venom metalloproteinase lebetase-4	*Macrovipera lebetina*	SVMP	24	19.4	-	-	-	-	0.008	12.4	0.091	19.4
146	Zinc metalloproteinase-disintegrin-like daborhagin-K	*Daboia russelii*	SVMP	69	6.2	-	-	-	-	-	-	0.112	6.2
147	Coagulation factor X-activating enzyme heavy chain	*Daboia siamensis*	SVMP	69	10.0	0.003	2.9	0.473	7.3	0.088	7.1	0.010	7.3
160	Coagulation factor X-activating enzyme heavy chain	*Macrovipera lebetina*	SVMP	68	11.4	0.013	5.4	1.243	11.4	0.105	6.5	0.020	5.4
166	Metalloproteinase F1	*Vipera ammodytes ammodytes*	SVMP	68	25.7	-	-	-	-	0.001	4.1	0.498	25.7
182	Antihemorrhagic factor cHLP-A	*Gloydius brevicaudus*	SVMP	36	4.0	-	-	-	-	-	-	0	4.0
184	Zinc metalloproteinase/disintegrin	*Macrovipera lebetina*	SVMP	53	11.9	-	-	0.008	4.8	0.027	8.8	0.657	7.9
185	Zinc metalloproteinase-disintegrin-like VLAIP-B	*Macrovipera lebetina*	SVMP	68	9.3	0	4.6	-	-	-	-	0.052	6.5
186	Zinc metalloproteinase-disintegrin-like VLAIP-A	*Macrovipera lebetina*	SVMP	68	25.2	0.005	17.7	0.138	14.9	0.110	17.5	0.048	19.0
192	Group III snake venom metalloproteinase	*Echis ocellatus*	SVMP	69	10.7	-	-	0	7.9	0	10.7	-	-
194	Zinc metalloproteinase-disintegrin-like bothrojarin-2	*Bothrops jararaca*	SVMP	24	11.9	-	-	0.015	11.9	0.017	11.9	0.089	11.9
1	Renin-like aspartic protease	*Echis ocellatus*	TBP	43	9.4	0.009	4.6	0.040	4.8	0.039	4.8	0.055	4.8
8	Aminopeptidase A	*Gloydius brevicaudus*	TBP	110	7.6	0	2.1	0.004	2.5	0.007	2.5	0.029	7.6
20	Aminopeptidase N	*Gloydius brevicaudus*	TBP	106	1.3	-	-	-	-	-	-	0	1.3
68	Glutaminyl-peptide cyclotransferases	*Daboia russelii*	TBP	42	37.8	0.109	37.8	0.092	29.1	0.055	32.1	0.085	37.8
111	Glutaminyl-cyclase	*Ovophis okinavensis*	TBP	40	33.2	0.012	33.2	-	-	-	-	-	-
120	Cathepsin D	*Ophiophagus hannah*	TBP	30	16.3	0.013	16.3	-	-	-	-	-	-
121	Endoplasmic reticulum aminopeptidase 1	*Ophiophagus hannah*	TBP	91	1.6	0.003	1.6	-	-	-	-	-	-
123	Renin	*Ophiophagus hannah*	TBP	40	5.5	0.016	2.2	0.059	5.5	0.048	5.5	0.023	5.5
149	Renin	*Echis coloratus*	TBP	12	23.9	-	-	-	-	0.013	23.9	-	-
167	Xaa-Pro aminopeptidase 2	*Boiga irregularis*	TBP	76	25.7	0.420	25.7	0.041	17.3	0.114	23.6	0.042	17.3
178	Peptidyl-prolyl cis-trans isomerase	*Crotalus adamanteus*	TBP	22	12.9	0.006	12.9	-	-	-	-	-	-
204	Dipeptidase 2-like	*Thamnophis sirtalis*	TBP	33	7.4	0.002	7.4	-	-	0.003	7.4	0.019	7.4
207	Xaa-Pro aminopeptidase 2-like	*Thamnophis sirtalis*	TBP	27	19.4	0.057	19.4	0.011	11.3	0.016	19.4	-	-
60	Snake venom vascular endothelial growth factor toxin vammin	*Vipera ammodytes ammodytes*	VEGF	16	49.7	5.315	31.0	4.239	44.1	5.109	36.6	2.446	32.4
88	Snake venom vascular endothelial growth factor toxin HF	*Vipera aspis aspis*	VEGF	12	65.5	0.083	65.5	0.002	58.2	0.002	48.2	-	-
148	Vascular endothelial growth factor A	*Echis coloratus*	VEGF	22	34.4	0.017	34.4	-	-	0.004	17.2	-	-
150	Vascular endothelial growth factor F	*Echis coloratus*	VEGF	16	28.5	-	-	0.390	28.5	0.640	28.5	0.030	18.8

^1^ B-NAP: Bradykinin potentiating and C-type natriuretic peptides; BP: Blood protein; CP: Cysteine Proteases; CRISP: Cysteine-rich secretory protein; CTL: C-type lectin like; Dis: Disintegrin; Hya: Hyaluronidase; Kunitz: Kunitz type proteinase inhibitor; LAAO: l-amino acid oxidase; NGF: Nerve growth factor; Nuc: Nucleic acid degrading enzymes; OP: Other protein; PLA2: Phospholipase A_2_; PLB: Phospholipase B; SP: Serine proteinase; SVMP: Metalloproteinase; TBP: Toxin biosynthesis proteins (including aminopeptidases); VEGF: Vascular endothelial growth factor.

**Table 2:** Protein families found in the venoms of Russian vipers.

Protein Family ^3^	# of Identified Proteins	Protein Abundance ^1^ LFQ/INT, % (# of Identified Proteins ^2^)			
		*V. nikolskii*	*V. kaznakovi*	*V. orlovi*	*V. renardi*
PLA2	(29)	64.68/65.96 (14)	41.03/36.43 (11)	24.21/27.27 (14)	44.05/47.64 (18)
SVMP	(32)	0.66/0.66 (8)	16.15/16.31 (19)	14.77/13.92 (21)	11.98/10.53 (28)
CTL	(18)	4.01/3.9 (9)	12.48/13.44 (15)	11.2/9.46 (15)	3.46/3.24 (8)
SP	(27)	19.34/20.51 (20)	10.79/10.96 (18)	23.97/22.61 (19)	7.87/6.03 (15)
CRISP	(7)	0.66/0.41 (2)	9.72/10.89 (7)	12.2/12.3 (7)	7.98/8.26 (4)
LAAO	(11)	0.08/0.07 (5)	3.99/4.33 (8)	4.59/4.19 (10)	4.21/3.56 (8)
VEGF	(4)	7.57/5.42 (3)	3.96/4.63 (2)	4.2/5.76 (4)	2.92/2.48 (2)
Dis	(5)	0/0 (0)	0.53/0.52 (4)	0.56/0.59 (3)	13.43/14.04 (5)
OP	(28)	0.17/0.28 (11)	0.49/1.13 (13)	0.95/0.96 (16)	1.8/2.15 (8)
PLB	(4)	0.12/0.1 (2)	0.32/0.34 (3)	0.52/0.5 (3)	0.54/0.51 (4)
Nuc	(7)	0.88/0.92 (6)	0.21/0.6 (4)	2.12/1.73 (5)	0.47/0.32 (3)
TBP	(13)	0.68/0.65 (11)	0.17/0.25 (5)	0.3/0.3 (8)	0.33/0.25 (7)
NGF	(2)	0.33/0.28 (1)	0.14/0.12 (2)	0.25/0.25 (1)	0.12/0.09 (1)
Hya	(2)	0/0.01 (2)	0.01/0.02 (2)	0/0.01 (2)	0/0 (0)
BP	(14)	0.15/0.12 (12)	0/0.01 (2)	0.01/0.01 (3)	0.06/0.1 (9)
B-NAP	(1)	0.01/0.01 (0)	0/0.03 (1)	0/0.01 (1)	0/0 (0)
Kunitz	(5)	0.66/0.7 (4)	0/0 (0)	0.15/0.12 (3)	0.79/0.8 (4)
CP	(1)	0/0 (1)	0/0 (0)	0/0 (0)	0/0 (0)
total	(210)	(111)	(116)	(135)	(124)

^1^ Protein abundance was calculated on the basis of peptide abundances for the peptides identified by MS/MS, as well as the peptides identified by MS1 matching between chromatograms. Protein abundances were calculated either on the basis of the MaxLFQ (Label-Free Quantification) algorithm (LFQ) or on the basis of the comparison of total protein intensities (sums of peptide intensities were calculated for each protein) within a single venom (INT). ^2^ Numbers of proteins were calculated on the basis of the peptides identified by MS/MS only (no MS1 matching hits were used). Therefore the protein might not be listed as identified, but it would still be quantified ”By Matching” with non-zero abundance value (e.g., B-NAP in *V. nikolskii* venom). ^3^ B-NAP: Bradykinin potentiating and C-type natriuretic peptides; BP: Blood protein; CP: Cysteine Proteases; CRISP: Cysteine-rich secretory protein; CTL: C-type lectin like; Dis: Disintegrin; Hya: Hyaluronidase; Kunitz: Kunitz type proteinase inhibitor; LAAO: l-amino acid oxidase; NGF: Nerve growth factor; Nuc: Nucleic acid degrading enzymes; OP: Other protein; PLA2: Phospholipase A_2_; PLB: Phospholipase B; SP: Serine proteinase; SVMP: Metalloproteinase; TBP: Toxin biosynthesis proteins (including aminopeptidases); VEGF: Vascular endothelial growth factor.

**Table 3:** Snake venom protein families for which peptides were found in peptidome only.

Family	Number of Proteins	Number of Peptides
CTL	4	5
Dis	3	7
Kunitz	2	2
LAAO	2	3
NAP	6	14
PLA2	1	1
SP	2	2
SVMP	11	22
VEGF	1	1

**Table 4:** Snake venom protein families represented in viper venoms.

Snake Venom Protein Family	*V. kaznakovi* Venom	*V. renardi* Venom	*V. orlovi* Venom	*V. nikolskii* Venom	*V.* *anatolica* Venom ^1^	*V. raddei* Venom ^2^	*V. a. ammodytes* Venom ^3^	*V. a. meridionalis* Venom ^3,4^
PLA2	+	+	+	+	+	+	+	+ ^3^
SP	+	+	+	+	+	+	+	+ ^3^
Dis	+	+	+	−	+	+	+	+ ^3^
CRISP	+	+	+	+	+	+	+	−
Kunitz	−	+	+	+	+	+	−	+ ^4^
LAAO	+	+	+	+	−	+	+	+ ^3^
SVMP	+	+	+	+	+	+	+	+ ^3^
NGF	+	+	+	+	−	+	+	+ ^3^
CTL	+	+	+	+	+	+	−	−
PLB	+	+	+	+	−	−	−	−
VEGF	+	+	+	+	−	+	+	+ ^3^
Nuc	+	+	+	+	−	−	−	−
B-NAP	+	+	+	+	−	+	−	+ ^4^
Hya	+	−	+	+	−	−	−	−

^1^ Taken from [13]; ^2^ Taken from [14]; ^3^ Taken from [15]; ^4^ Taken from [34].

## Supplementary Materials

The following materials are available online at www.mdpi.com/2072-6651/8/4/105/s1, Table S1: The detailed list of proteins identified in Russian viper venoms; Table S2: The detailed peptide identification list for proteome results (contains all peptide-related data from MaxQuant proteome analysis); Table S3: Endogenous peptides found in four viper venoms: “endogPeptidesWithProteins”—combined peptide and protein data, “endogenousPeptides”—peptide only data, “endogenousPeptidogenicProteins”—protein only data; Table S4: Endogenous peptides identified in proteins unique for peptidome analysis.

## Abbreviations

The following abbreviations are used in this manuscript:

FC: Fold Changes
FDR: False Discovery Rate
B-NAP: Bradykinin potentiating and C-type natriuretic peptides
BP: Blood protein
CP: Cysteine Proteases
CRISP: Cysteine-rich secretory protein
CTL: C-type lectin like
Dis: Disintegrin
Hya: Hyaluronidase
Kunitz: Kunitz type proteinase inhibitor
LFQ: label-free quantification
LAAO: l-amino acid oxidase
NGF: Nerve growth factor
Nuc: Nucleic acid degrading enzymes
OP: Other protein
PLA2: Phospholipase A_2_
PLB: Phospholipase B
SP: Serine proteinase
SVMP: Snake venom metalloproteinase
TBP: Toxin biosynthesis proteins (including aminopeptidases)
VEGF: Vascular endothelial growth factor
